# 

**Published:** 1892-04-09

**Authors:** 


					'* \XlU
N price twopence.
Vo1' XH.?April 9th, 1892.
tranj?f_a^ the General PoBt Office as a Newspaper for
l8?on in the United Kingdom and Abroad.]
Apbh. 9th, 18S
WITH EXTRA NURSING SUPPLEMENT.
Per Annum, 8s. 6d., post free.
Monthly Farts, 6d.; post free, 8d.
Ditto per Annnrn, 8s., post free.
No. 289.' Vol. XII.] Saturday,
Aprii 9th, 1892.
[Twopence.

				

